# Development and Validation of Risk Assessment Model for Pelvic Organ Prolapse Based on A Retrospective Study with Machine Learning Algorithms

**DOI:** 10.1007/s00192-025-06046-9

**Published:** 2025-02-03

**Authors:** Ling Mei, Linbo Gao, Tao Wang, Dong Yang, Weixing Chen, Xiaoyu Niu

**Affiliations:** 1 Department of Obstetrics and Gynecology, West China Second University Hospital, Sichuan University, Chengdu, 610041 China; 2 Center of Translational Medicine, Key Laboratory of Birth Defects and Related Diseases of Women and Children, West China Second University Hospital, Sichuan University, Chengdu, 610041 China; 3 Laboratory of Metabolomics and Gynecological Disease Research, West China Second University Hospital, Sichuan University, Chengdu, 610041 China; 4 Department of Data Science, Guangzhou AID Cloud Technology Co., Ltd, Guangzhou, 510663 China

**Keywords:** Machine learning, Pelvic organ prolapse, Risk assessment model, Shapley additive explanations method

## Abstract

**Introduction and Hypothesis:**

We aimed to develop and validate a clinically applicable risk assessment model for identifying women at a high risk of pelvic organ prolapse (POP) based on a retrospective practice.

**Methods:**

This study enrolled patients with and without POP between January 2019 and December 2021. Clinical data were collected and machine learning models were applied, such as multilayer perceptron, logistic regression, random forest (RF), light gradient boosting machine and extreme gradient boosting. Two datasets were constructed, one comprising all variables and the other excluding physical examination variables. Two versions of the machine learning model were developed. One was for professional doctors, and the other was for community-health providers. The area under the curve (AUC) and its confidence interval (CI), accuracy, F1 score, sensitivity, and specificity were calculated to evaluate the model’s performance. The Shapley Additive Explanations method was used to visualize and interpret the model output.

**Results:**

A total of 16,416 women were recruited, with 8,314 and 8,102 in the POP and non-POP groups respectively. Eighty-seven variables were recorded. Among all candidate models, the RF model with 13 variables showed the best performance, with an AUC of 0.806 (95% CI 0.793–0.817), accuracy of 0.723, F1 of 0.731, sensitivity of 0.742, and specificity of 0.703. Excluding the physical examination variables, the RF model with 11 variables showed an AUC, accuracy, F1 score, sensitivity, and specificity of 0.716, 0.652, 0.688, 0.757, and 0.545 respectively.

**Conclusions:**

We constructed a clinically applicable risk warning system that will help clinicians to identify women at a high risk of POP.

**Supplementary Information:**

The online version contains supplementary material available at 10.1007/s00192-025-06046-9

## Introduction

Pelvic organ prolapse (POP) is the descent of the anterior vaginal wall, posterior vaginal wall, uterus, cervix, or vaginal vault after hysterectomy and usually combines with cystocele, rectocele, or enterocele [1]. According to previous research, women in the USA have a 13% lifetime risk of undergoing surgery for POP [2]. The number of women experiencing POP in the USA is expected to increase by approximately 50% by 2050 [3]. POP is associated with many pelvic problems, such as urinary symptoms, sexual dysfunction, and anorectal symptoms, which can significantly affect a woman’s quality of life.

Notably, many risk factors for POP have been identified, including obesity, tobacco use, certain physical and sports activities, lifting and carrying heavy loads, defecation or urinary pushing, and chronic coughing. These factors are associated with lifestyle and can be modified. Other factors that cause the denervation of the pelvic floor, such as physiological age, gynecological and obstetrical history, hormonal status, and neurological disorders, are nonmodifiable. So far, management has often targeted the factors that could be modified, such as lifestyle, pelvic floor muscle training, pessary placement, topical estrogen administration, and reconstructive surgery [4, 5].

Despite the discovery of many risk factors, not all individuals with risk factors develop POP. Therefore, identifying high-risk populations for POP based on risk factors alone and conducting early interventions is impossible. Conversely, many factors may be associated with POP but have not been fully recognized. For example, a study indicated that women with a larger genital hiatus (GH) are more likely to develop prolapse more rapidly. Specifically, the estimated median time to develop prolapse for women with a GH of 3 cm is 33.4 years compared with 5.8 years for women with a GH of ≥ 4.5 cm [6]. A study compared the pelvic computed tomography (CT) scan images of women with POP and control women and found that women with POP had a significantly larger anterior pelvic area (*p* < 0.05), a considerably longer interspinous diameter (*p* < 0.05), a significantly longer distance from the ischial spine to the pubic symphysis (*p* < 0.05), and a significantly longer pelvic outlet (*p* < 0.05) than the controls [7]. A few studies [8, 9] have predicted the first occurrence of POP, and none are based on population data. Therefore, in this study, we aimed to construct a clinically applicable warning system for identifying women at a high risk of POP using machine learning models based on data from a pelvic floor disease surveillance database. This helps to conduct earlier interventions, delay disease progression, and reduce the prevalence of POP.

## Materials and Methods

### Study Design and Participants

This retrospective case–control study was conducted at our hospital, and participants were recruited from the Postpartum Clinic and Rehabilitation Center of Pelvic Diseases. Data from patients between January 2019 and December 2021 were extracted from the hospital information system. Women aged < 18 years old were excluded. Women with Pelvic Prolapse Quantification ≥ stage II with or without POP symptoms were all included as the POP group, and women with Pelvic Prolapse Quantification ≤ stage I were included as the non-POP group.

### Data Collection and Variable Selection

The following data were collected:

1. Demographic characteristics, such as age, educational background, height, body weight, body mass index (BMI), cigarette smoking, and alcohol consumption.
2. History of pregnancy and childbirth, such as number of vaginal deliveries, cesarean sections, ectopic pregnancies, abortions, and total number of pregnancies.
3. Past medical history, such as hypertension, diabetes mellitus, hepatitis, and menstrual cycle.
4. History of present illnesses, such as urination-related, vaginal bleeding, or liquid leakage, dizziness, visual blurring, sleep disorders, and constipation.
5. Physical examination, including the POP Quantification grading system, (point Aa, point Ba, point Ap, point Bp, length of GH, length of perineal body, and total vaginal length (TVL), manual manometry of pelvic muscles (including fiber I-rich muscles and fiber II-rich muscles, which are specialized for continuous activity and phasic activity respectively), and vaginal laxity.
6. Surgical history, including episiotomy during delivery, hysterectomy, cervical conization, cesarean section directly without labor, cesarean section during vaginal birth, and myomectomy.
7. Family history of POP and urinary incontinence (UI).

For missing variables, mean imputation was used to impute the missing continuous variables, and mode imputation was used to impute the missing discrete data. The missing data rate is shown in Appendix 1. We first applied preliminary feature screening among the recorded variables by excluding variables with *p* values ≥ 0.05 when comparing the POP and non-POP groups. Recursive feature elimination techniques were used for delicate feature selection.

### Statistical Analysis

The event per variable (EPV) rule was adopted to ensure that our sample size was sufficiently large to create a prediction model [10]. When developing prediction models for binary or time-to-event outcomes, an established rule of thumb for the required sample size is used to ensure that each predictor parameter includes at least ten events in the prediction model equation. Our study included 16,416 women, including 8314 with POP and an initial 87 variables, which made the EPV significantly > 10.

Data cleaning was performed using Python (Anaconda Distribution version 3.7), package NumPy (version 1.21.5), and Pandas (version 1.4.2). The Scikit-learn (version 1.1.1) library was used to develop the machine learning models. Five machine learning models, including multilayer perceptron, logistic regression, RF, light gradient boosting machine, and extreme gradient boosting, were used to estimate the predictive value of POP. The area under the curve (AUC), confidence interval (CI), accuracy, F1, sensitivity, and specificity were calculated. Categorical variables were expressed as numbers and percentages and compared between the two groups using the Chi-squared or Fisher’s exact test. The mean with standard deviation was expressed and compared for continuous variables using the independent-sample Student’s* t* test for normally distributed samples and the Mann–Whitney *U* test for non-normally distributed samples. The dataset was randomly separated into a 70% training set to establish the predictive models and a 30% testing set to validate the model performance. The K-means method was used to cluster data into clusters of low-, moderate-, and high-risk levels to determine the optimal cutoff value based on the predicted risk. Notably, multiple choices could be set as the number of clusters for the K-means method; however, we used three levels to ensure convenience in the clinical application. The Shapley Additive Explanations (SHAP) method was applied to visualize and interpret the model outputs using the Python SHAP library (version 0.41.0). A dataset devoid of physical examination variables was meticulously compiled to ensure the broader applicability of the predictive models. Machine learning algorithms were subsequently developed using this dataset to enhance the tool’s accessibility for family physicians and women, facilitating its future use in nonclinical settings.

## Results

### Baseline Features of the Participants

In total, 16,416 women were recruited; 8314 and 8102 were in the POP and non-POP groups respectively, and 87 variables were recorded, as shown in Appendix 2. The clinical features of the study population are summarized in Table 1. Compared with the non-POP group, the POP group was older. They had a lower level of education, a higher BMI, a higher number of gestations, parities, and abortions, and a higher frequency of UI, defecation dysfunction, vaginal laxity, and chronic diseases such as hypertension, diabetes mellitus, and hemorrhoids. Delivery factors such as vaginal delivery, assisted vaginal birth, episiotomy, or perineal laceration during delivery were more frequent in the POP group. Family history of POP and UI are also risk factors for POP. Physical examinations revealed a longer GH, TVL, perineal body length, and lower pelvic floor muscle strength in the POP group. However, there were no significant differences in alcohol consumption, smoking status, birth weight of babies, and chronic cough between the two groups.

**Table 1:** Clinical features of participants

Clinical features	POP group (*n* = 8314)	Non-POP group (*n* = 8102)	*p* value
Age, years (SD)	30.61 (7.30)	29.47 (3.94)	< 0.001
Height, cm (SD)	160.53 (20.41)	160.84 (29.55)	0.276
Body mass index ≥ 24 kg/m^2^	3632 (44.14%)	3068 (38.24%)	< 0.001
Menopause	244 (2.93%)	77 (0.95%)	< 0.001
Educational background			< 0.001
Below primary school	22 (0.26%)	12 (0.15%)	
Primary school	377 (4.54%)	189 (2.33%)	
Middle school	4652 (55.96%)	3332 (41.14%)	
High school	168 (2.02%)	193 (2.38%)	
Undergraduate	688 (8.28%)	1002 (12.37%)	
Bachelor degree	1729 (20.80%)	2470 (30.50%)	
Master degree or PhD	677 (8.14%)	901 (11.12%)	
Employment			
Physical worker	864 (10.39%)	567 (7.00%)	< 0.001
Intellectual worker	6896 (82.94%)	6658 (82.18%)	0.195
Physical and intellectual worker	552 (6.64%)	873 (10.78%)	< 0.001
Lifestyle-related			
Alcohol drinking	12 (0.16%)	17 (0.23%)	0.345
Smoking	23 (0.28%)	23 (0.28%)	0.93
Pregnancy-related			
Number of gestations (SD)	2.12 (1.31)	1.99 (1.22)	< 0.001
Number of deliveries (SD)	1.35 (0.56)	1.25 (0.45)	< 0.001
Number of induced labors (SD)	0.20 (0.55)	0.19 (0.51)	0.039
Number of abortions (SD)	0.75(1.04)	0.70 (0.99)	0.024
Preterm labor	221 (2.66%)	418 (5.16%)	< 0.001
Term labor	3285 (39.51%)	4218 (52.06%)	< 0.001
Number of vaginal deliveries (SD)	0.26 (0.58)	0.08 (0.29)	< 0.001
Number of caesarean sections			< 0.001
0	6547 (88.12%)	6192 (83.93%)	
1	842 (11.33%)	1138 (15.42%)	
2	36 (0.48%)	48 (0.65%)	
3 and more	5 (0.07%)	0 (0%)	
Birth-related			
Forceps/vacuum-assisted birth	259 (4.25%)	183 (2.89%)	< 0.001
Episiotomy during delivery	2094 (25.19%)	1158 (14.29%)	< 0.001
Vaginal delivery	3412 (41.04%)	1655 (20.43%)	< 0.001
Cesarean section directly without labor	1680 (20.21%)	3239 (39.98%)	< 0.001
Cesarean section during trial of vaginal birth	664 (7.99%)	1256 (15.50%)	< 0.001
Perineal laceration during vaginal delivery	1862 (30.59%)	801 (12.67%)	< 0.001
Lateral episiotomy during delivery	2100 (34.51%)	1161 (18.37%)	< 0.001
Baby’s birth weight (SD)	3.41 (0.94)	3.44 (1.02)	0.17
Number of fetuses (SD)	1.33 (0.57)	1.23 (0.47)	< 0.001
Labor analgesia	329 (3.96%)	169 (2.09%)	< 0.001
Chronic condition			
Hemorrhoids	4431 (53.30%)	4063 (50.15%)	< 0.001
Chronic rhinitis	141 (1.70%)	236 (2.91%)	< 0.001
Chronic cough	144 (1.73%)	118 (1.46%)	0.159
Hepatitis	2448 (32.95%)	2448 (32.95%)	< 0.001
Hypertension	146 (1.76%)	85 (1.05%)	< 0.001
Diabetes mellitus	155 (1.86%)	115 (1.42%)	0.025
Uterine adenomyosis	162 (1.95%)	205 (2.53%)	0.012
History of hysterectomy	35 (0.42%)	12 (0.15%)	< 0.001
Defecation			
Normal fecus	7336 (99.28%)	7313 (99.55%)	0.033
Constipation	2969 (35.72%)	2740 (33.83%)	0.011
Urination			
Normal urination	7245 (99.74%)	7317 (99.96%)	< 0.001
Urge urinary incontinence	44 (0.53%)	16 (0.20%)	< 0.001
Stress urinary incontinence	561 (6.75%)	111 (1.37%)	< 0.001
Mixed urinary incontinence	19 (0.23%)	6 (0.07%)	0.011
Urinary incontinence during pregnancy	1238 (14.96%)	868 (10.76%)	< 0.001
Postpartum urinary incontinence	779 (9.41%)	311 (3.86%)	< 0.001
Urinary incontinence before pregnancy	175 (2.11%)	96 (1.19%)	< 0.001
Urinary incontinence	410 (4.93%)	236 (2.91%)	< 0.001
Vagina-related			
Vaginal bleeding or liquid leaking	2289 (30.73%)	2086 (27.63%)	< 0.001
Dysmenorrhea	1355 (20.75%)	1821 (27.05%)	< 0.001
Vaginal laxity	4423 (53.20%)	2215 (27.34%)	< 0.001
Tissue prolapse from the vagina	205 (2.47%)	4 (0.05%)	< 0.001
Old lacerations	109 (1.31%)	7 (0.09%)	< 0.001
Length of perineal body (SD)	3.50 (0.79)	3.44 (0.68)	< 0.001
Total vaginal length (SD)	7.31 (1.19)	7.29 (1.15)	< 0.001
Length of genital hiatus (SD)	3.14 (0.87)	2.78 (0.71)	< 0.001
Muscle-related			
Normal vaginal muscle strength	2568 (30.89%)	3452 (42.61%)	< 0.001
Manual manometry of superficial type II fiber-rich muscles (SD)	1.61 (0.68)	1.65 (0.69)	< 0.001
Manual manometry of deep type II fiber-rich muscles (SD)	1.60 (0.68)	1.65 (0.69)	< 0.001
Manual manometry of superficial type I fiber-rich muscles (SD)	1.36 (0.60)	1.39 (0.60)	0.013
Manual manometry of deep type I fiber-rich muscles (SD)	1.35 (0.60)	1.39 (0.60)	0.009
Family history-related			
Urinary incontinence	408 (6.39%)	323 (4.94%)	< 0.001
POP	409 (4.92%)	323 (3.99%)	0.004

*POP* pelvic organ prolapse, *SD* standard deviation

### Establishment of Pelvic Organ Prolapse Risk Assessing Models for Specialists

**Fig. 1:**
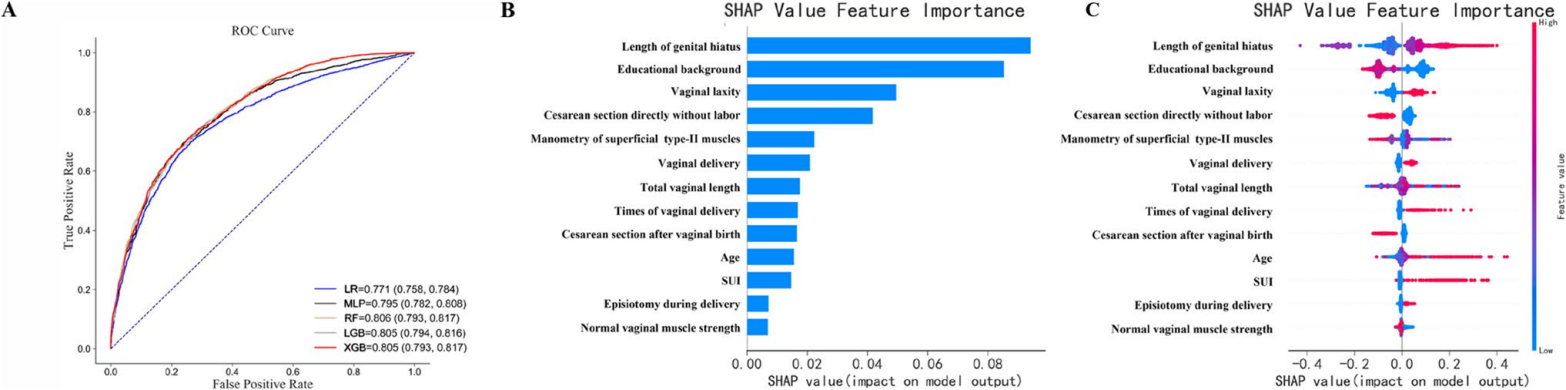
Receiver-operating characteristic (ROC) curves of machine learning models for predicting pelvic organ prolapse (POP) and each variable’s Shapley Additive Explanations (SHAP) value based on the random forest (RF) model. **A** ROC curves of machine learning models for predicting POP with a complete internal dataset. **B** Summary plot of SHAP showing the effect of each variable on the SHAP model output. The horizontal coordinate is the feature’s importance value, and the larger the value, the more important the variable is in the model prediction. **C** Correlation dot plot showing the effect of each variable on the SHAP model output. The red dot is concentrated on the right, meaning that the variable correlates positively with the outcome. The greater the value of the variable, the greater the contribution to the model prediction and the greater the possibility of the prediction being positive. *LR* logistic regression, *MLP* multilayer perceptron, *LGB* light gradient boosting, *XGB* extreme gradient boosting, *SHAP* SHapley Additive exPlanations

Pelvic organ prolapse risk-assessing models were established based on the complete clinical dataset presented in Appendix 3. In total, 11,491 and 4925 samples were used as the training and validation sets respectively. Thirteen variables were included in the predictive model. The RF model showed the best predictive performance, with an AUC of 0.806 (95% CI 0.793–0.817), accuracy of 0.723 (95% CI 0.709–0.735), F1 of 0.731 (95% CI 0.716–0.744), sensitivity of 0.742 (95% CI 0.724–0.758), and specificity of 0.703 (95% CI 0.684–0.722) (Fig. 1A).

The significance of the included feature variables was assessed using SHAP. Figure 1B and C show that the GH, educational background, vaginal laxity, direct cesarean section without labor, and manual manometry of superficial type II fiber-rich muscles ranked among the top five important features in significance. Longer GH, lower level of education, more severe vaginal laxity, weaker superficial type II fiber-rich muscles, longer vaginal length, and more vaginal deliveries are associated with the occurrence of POP. Episiotomy, vaginal delivery, and stress UI (SUI) are risk factors for POP, whereas cesarean section protects women from POP. Physical examination variables associated with pelvic floor disorders were included; therefore, the model is suitable for professional doctors of pelvic floor dysfunctional diseases.

**Fig. 2:**
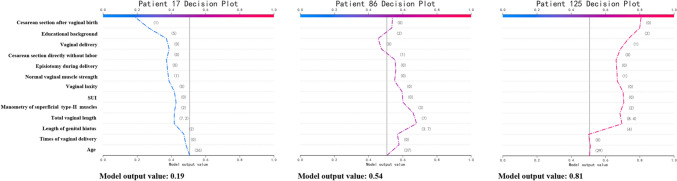
The model output value of the professional version for individual prediction. Three representative examples, namely participant numbers 17, 86, and 125. The important features of all predictors are shown for each participant. *SUI* stress urinary incontinence

According to the K-means clustering performed on the probability values of the training set, the risk index of POP ranged from 0 to 1, with 0–0.31 indicating low risk, 0.32–0.58 indicating moderate risk, and 0.59–1 indicating high risk. The SHAP plots of three individuals are presented to explain the model. As shown in Fig. 2, the data for three women (numbers 17, 86, and 125) were correctly classified. The decision plot contains the major factors for each individual’s final model output. Participant number 17, clinically diagnosed as “non-POP,” was calculated as having a low probability of POP. Patient number 86, clinically diagnosed as “POP,” was calculated as having a moderate probability of POP, and patient number 125, clinically diagnosed as “POP,” was calculated to have a high probability.

### Establishment of Assessing Models of Pelvic Organ Prolapse Risk for the General Public

**Fig. 3:**
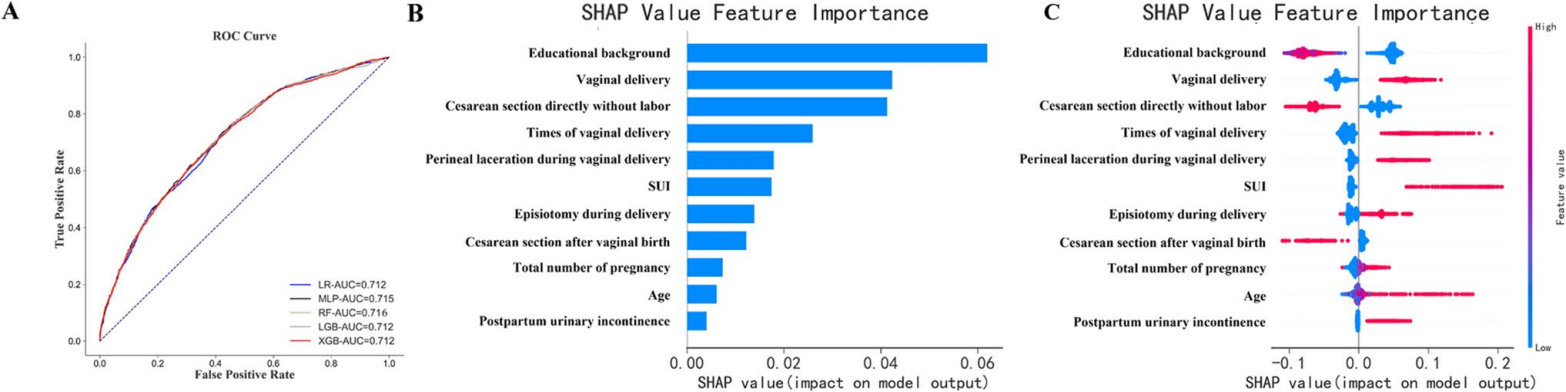
Receiver-operating characteristic (ROC) curves of machine learning models for predicting pelvic organ prolapse (POP) and each variable’s Shapley Additive Explanations (SHAP) value based on the random forest (RF) model with the dataset excluding physical examination variables. **A** ROC curves of machine learning models for predicting POP with the dataset excluding physical examination variables. **B** Summary plot of SHAP showing the effect of each variable on the SHAP model output. The horizontal coordinate is the feature’s importance value. **C** Correlation dot plot showing the effect of each variable on the SHAP model output. The red dot is concentrated on the right, meaning that the variable correlates positively with the outcome. *LR* logistic regression, *MLR* multilayer perceptron, *LGB* light gradient boosting machine, *XGB* extreme gradient boosting, *SUI* stress urinary incontinence

For the general public to conveniently use the model, a community version was constructed using a dataset that excluded 11 physical examination variables. In total, 11,491 and 4925 samples were used as training and validation sets respectively. As shown in Fig. 3A, the RF model with 11 variables had the best predictive performance, with an AUC of 0.716 (95% CI 0.701–0.729), accuracy of 0.652 (95% CI 0.639–0.665), F1 of 0.688 (95% CI 0.674–0.702), sensitivity of 0.757 (95% CI 0.739–0.774), and specificity of 0.545 (95% CI 0.523–0.564).

According to the SHAP summary plot, the top five important features in the RF model were educational background, vaginal delivery, direct cesarean section without vaginal delivery trial, number of vaginal labors, and perineal laceration during vaginal delivery (Fig. 3B and C). Women with a lower educational level, history of vaginal delivery, and perineal laceration were more likely to develop POP. Furthermore, direct cesarean section without trial of vaginal delivery protects women from POP.

**Fig. 4:**
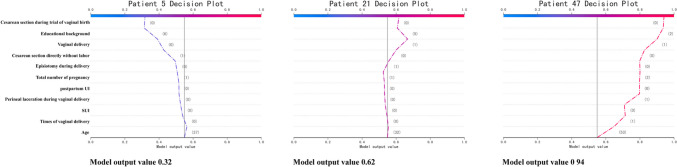
The model output value of the community version for individual prediction. Three representative examples, namely participant numbers 5, 21, and 47. The important features of all predictors are shown for each participant. *SUI* stress urinary incontinence

According to the K-means clustering performed on the probability values of the training set, the risk index of POP ranged from 0 to 1, with 0–0.42 indicating low risk, 0.43–0.64 indicating moderate risk, and 0.65–1 indicating high risk. The SHAP plots of the three individuals are presented to explain the model. Figure 4 shows that the data for the three samples (numbers 5, 21, and 47) were correctly classified. Participant number 5, clinically diagnosed as “non-POP,” was calculated as having a low probability of POP. Patient number 21, clinically diagnosed as “non-POP,” was calculated as having a moderate probability of POP, and patient number 47, clinically diagnosed as “POP,” was calculated as having a high probability.

### Geographical Multicenter External Validation of the Community Version

A geographical multicenter external validation was performed based on the dataset, excluding physical examination variables, in 22 hospitals between October 2022 and March 2023. In addition to our hospital, the other hospitals are located in different districts or cities in Sichuan Province, including tertiary and secondary hospitals. In total, 1658 participants were selected, including 757 women without POP and 901 women with POP. The RF model showed the best performance, with an AUC of 0.722. The community version of the POP predictive model can now be obtained from the website (https://analysis.aidcloud.cn/cn/predict/#/pop)*.*

## Discussion

Previous studies have mainly focused on the risk factors for POP or the predictors of POP recurrence after an initial surgery [11–15]. However, in the current study, we constructed two models for identifying women at a high risk of POP based on the complete pelvic medical records of 16,416 women. Based on a large sample size, machine learning techniques were used to construct a predictive model with 13 variables, with an AUC of 0.806 and accuracy of 0.723. Urogynecologists can use this model to assess the risk of POP. When specialized physical examination variables were excluded, 11 variables were included in the model, which showed the best overall performance, with an AUC of 0.716 and accuracy of 0.652. This model can help community or family doctors to rapidly assess the risk of POP, allowing them to offer appropriate health education to women at different risk levels.

Conventionally, multiple vaginal deliveries and aging are high-risk factors for POP; however, the most significant risk factors for the occurrence of POP are GH length, educational background, and vaginal laxity. The strongest risk factor in the model for experts was GH length, which was consistent with the results of a previous report [16] that found that women with a GH of ≥ 3.5 cm were nine times more likely to develop prolapse than those with a GH of ≤ 2.5 cm. A cross-sectional study [17] compared POP-Q scores of 100 women with and without apical prolapse. The GH > 4.5 cm was significantly associated with apical prolapse. A prospective study [18] indicated that the cumulative probability of prolapse increased substantially with increasing GH. The estimated median time to develop prolapse would be 5.8 years for a GH of ≥ 4.5 cm. Vaginal delivery is a well-known risk factor for POP. This is because the process impairs the pelvic floor muscles and fascia, leading to increased GH. In contrast, elective cesarean section is a preventive measure for reducing the occurrence of POP. We cannot vigorously promote elective cesarean sections and abolish vaginal birth; however, we should at least try to protect the integrity of the perineal body, avoid perineal incision without indications during vaginal delivery, suture the muscles and fascia layer-by-layer during perineal laceration repair, and restore the anatomical integrity of the pelvic floor, which are significant in preventing POP.

We also found that education level was negatively associated with the risk of POP. This is because women with a higher level of education may have better health awareness, healthier habits, and fewer chances of heavy physical labor, which helps to prevent POP. Other research has also indicated that women with a higher education level may have adequate knowledge about pelvic floor dysfunctions, which could lead to earlier treatment and improved symptoms and quality of life [19].

In our study, vaginal laxity was the third most common risk factor for POP. Notably, some experts consider vaginal laxity an early manifestation of POP, and early intervention is recommended, such as radiofrequency, carbon dioxide fractional laser, and surgery [20]. Human pelvic floor muscles are composed of the anal sphincter, musculus levator ani, urethral sphincter, and other muscle groups that are mainly divided into type I and type II muscle fibers [21]. Studies have shown that in patients with SUI, the mean values of systolic potential, strength, and time of type II muscle fibers after delivery are significantly reduced, which significantly impacts the occurrence of SUI. This indicates that type II muscle strength is abnormal during SUI, and the lesion appears earlier than that of type I muscle fibers [22]. In our model, type II muscle fiber strength was associated with POP, indicating that type II muscle fiber strength may be a more sensitive index of POP than type I muscle fiber strength.

Our study also found that SUI was a risk factor for POP, which is consistent with the results of other studies. According to epidemiological data, 30–80% of patients with POP report concurrent SUI [23]. POP and SUI are thought to develop following pelvic nerve, muscle, and connective tissue trauma. The threshold of neuromuscular compromise for symptomatic SUI is lower than that for POP [24].

A meta-analysis [25] was aimed at assessing risk factors for POP recurrence following colpocleisis and recruited 954 studies, which found that postoperative TVL was significantly longer in the recurrence group. Our study also found that TVL was positively associated with the occurrence of POP. Oh et al. [26] indicated that descent of the vaginal apex beyond the halfway point of the vagina could be considered an anatomical threshold for clinically relevant apical prolapse. It can be interpreted as a patient with a TVL of 8 cm, when the C point is −4 cm, already indicating apical prolapse, which is easier than the patient with the TVL of 6 cm whose C point needs to reach −3 cm. Similarly, according to the International Continence Society (ICS) definition, apical prolapse means descent of the vaginal cuff scar or cervix, below a point that is 2 cm less than the TVL; thus, the longer TVL, the more easily C reaches the point.

To make the model more convenient for clinical and family applications, we developed an online calculator using features that are easily accessible to community health care providers and women themselves. Professional and community versions will provide early warnings for POP and indicate women at a high risk. In the follow-up cohort, women with moderate risk can be advised to adjust their lifestyle habits, engage in sports training, and present for follow-up once every year. Women at a high risk should also be advised to perform Kegel exercises for pelvic muscle training, recommended as the first-line therapy for POP [1, 4, 5]. Given that vaginal laxity is an early manifestation of POP, if women at a high risk have moderate or severe vaginal laxity, energy interventions such as radiofrequency or laser treatment, or surgery could be considered. So far, the evidence [27] indicates that sexual function in women with vaginal laxity who underwent radiofrequency and laser treatment improved in observational studies but not in randomized controlled trials. Improvement in pelvic floor muscle strength was observed in women with vaginal laxity after the intervention. Women should be referred to a higher-level hospital if the main complaints associated with POP are found during follow-up. They should undergo more precise and individualized surveillance and guidance to manage the disease.

## Conclusions

We established professional and community versions of predictive models for POP based on large datasets and machine learning algorithms. These models can screen women at different levels of risk of developing POP; therefore, other intervention strategies can be provided accordingly.

## Supplementary Information

Below is the link to the electronic supplementary material.

Supplementary file1 (XLSX 13 KB)

Supplementary file2 (XLSX 12 KB)

Supplementary file3 (XLSX 15 KB)

## Data Availability

The data used and/or analyzed during the current study are available from the corresponding author on reasonable request.

## Abbreviations

AUC: Area under the curve
CI: Confidence interval
GH: Genital hiatus
LGB: Light gradient boosting
LR: Logistic regression
MLP: Multilayer perceptron
POP: Pelvic organ prolapse
RF: Random forest
SD: Standard deviation
SHAP: Shapley Additive Explanations
UI: Urinary incontinence
XGB: Extreme gradient boosting
